# Left coronary sinus of valsalva aneurysm dissecting into interventricular septum: a case report

**DOI:** 10.1186/s13019-024-02513-4

**Published:** 2024-02-04

**Authors:** Lei Zhang, Dong-hui Ji, Xiao-yun Li, Kuo-peng Liang

**Affiliations:** Department of Ultrasound, Xingtai People’s Hospital, Xingtai, Hebei China

**Keywords:** Aneurysm, Coronary sinus, Valsalva, Interventricular septum

## Abstract

**Background:**

Sinus of Valsalva aneurysm (SVA) is an extremely rare condition, and its rupture causes acute symptoms such as chest pain and dyspnea. Ruptured SVA is frequently associated with other congenital defects.

**Case presentation:**

A 37-year-old male presented with SVA originating from the left coronary sinus that ruptured into the interventricular septum. SVA was diagnosed by echocardiography, cardiac computed tomography and magnetic resonance imaging, and confirmed during the operation.

**Conclusions:**

SVA is a rare cardiac abnormality which can lead to severe clinical symptoms upon rupture. Immediate surgery is necessary to repair the ruptured SVA.

## Background

Sinus of Valsalva aneurysm (SVA) is a rare cardiac anomaly, with an incidence of 0.1–3.5% among all congenital heart defects and a prevalence of 0.09% in the general population. In Antonio Maria Valsalva’s Opera, SVAs were first described in detail in 1740 [1]. SVA may be acquired or congenital and most commonly involves the right or non-coronary sinuses. Acquired SVA is less frequently reported than congenital SVA, making it more difficult to determine its incidence. Congenital aneurysms are more common and often caused by weakness at the junction of the aortic media and the annulus fibrous. This report describes a case of left coronary SVA that ruptured into the interventricular septum, leading to a large dissecting aneurysm. The left coronary SVA was diagnosed by echocardiography, cardiac computed tomography (CT) and magnetic resonance imaging (MRI).

### Case presentation

**Fig. 1:**
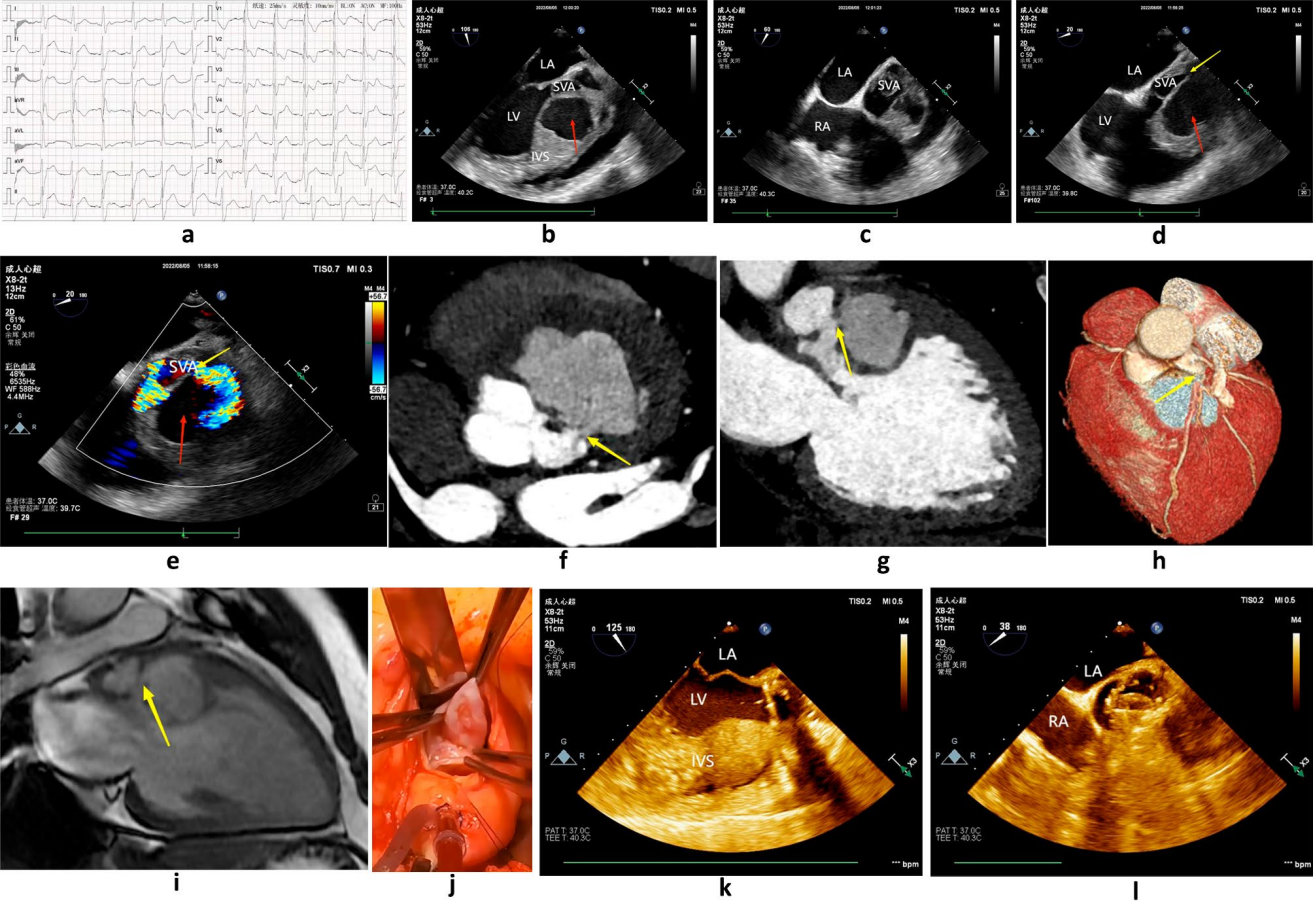
**a** Twelve-lead ECG with evidence of complete right bundle branch block. **b-c** TEE showed a large cystic formation in the interventricular septum (red arrow) and SVA originating from the left coronary sinus. **d** TEE showed a fistulous tract from the left sinus of Valsalva dissecting into the interventricular septum (yellow arrow). **e** A color Doppler ultrasound showed a shunt between the aneurysmatic pouch and the interventricular septum (yellow arrow). **f-i** Cardiac CT and MRI showed a fistulous tract from the left sinus of Valsalva dissecting into the interventricular septum (yellow arrow). **j** Operational findings confirmed SVA. **k-l** TEE showed that the disappeared ventricular septal dissection and the well-functioning mechanical aortic valve

A 37-year-old male was admitted to our clinic with complaints of chest pain and palpitations for three days. Physical examination revealed a systolic murmur at the upper left sternal border. The patient’s functional class was 2–3 according to the New York Heart Association (NYHA) classification. His electrocardiogram(ECG) showed complete right bundle branch block (Fig. 1a). Transthoracic echocardiography was performed, which showed severe aortic insufficiency and a large cystic formation in the interventricular septum. Transesophageal echocardiography (TEE) also showed a large cystic formation in the interventricular septum, which increased in size during diastole and decreased during systole. The SVA originated from the left coronary sinus (Fig. 1b-c). A fistulous tract was seen from the left sinus of Valsalva dissecting into the interventricular septum but did not communicate with the right ventricle. (Fig. 1d). Color Doppler ultrasound showed a shunt between the aneurysmatic pouch and the interventricular septum (Fig. 1e). Cardiac CT and MRI also revealed an aneurysm arising from the left coronary sinus of Valsalva and dissecting the interventricular septum (Fig. 1f-i). Given the severe aortic regurgitation and to mitigate the threat of aneurysm rupture, surgery was performed (Fig. 1j). We excised the cystic cavity and simultaneously performed a mechanical valve replacement of the aortic valve. After the surgery, TEE showed that the interventricular septal dissection had disappeared, and the mechanical function of the aortic valve was satisfactory (Fig. 1k-l).

## Discussion

SVA is a rare cardiac abnormality mainly occurring due to congenital dysplasia of the aortic sinus wall, lacking elastic fibers and smooth muscle tissue in the middle layer. Due to the aortic high-pressure blood flow, the sinus wall gradually expands and thins to the adjacent cardiac cavity to form a sinus aneurysm. In addition to innate factors, SVA can be secondary to infections (syphilis, bacterial endocarditis, or tuberculosis), trauma, or connective tissue diseases that affect the aortic wall [2]. SVA can be associated with other cardiac malformations, the most common of which are ventricular septal defects, bicuspid aortic valve malformations, and aortic regurgitation [3].

SVA mainly occurs in the right coronary sinus, accounting for more than 75% of the cases, followed by the non-coronary sinus, accounting for about 15%. It rarely occurs in the left coronary sinus and is associated with asymmetric stress distribution on the three aortic sinuses [4]. Owing to the anatomical relationship, right coronary SVA is usually adjacent to the right ventricular outflow tract and often ruptures into the right ventricle or right atrium. In contrast, the non-coronary SVA often ruptures into the right atrium [5]. The left coronary SVA invading the interventricular septum described in this report is extremely rare.

The hemodynamic changes caused by the ruptured SVA depend on the size and the compartments at both ends of the rupture. In general, the larger the rupture, the greater the shunt volume. When an SVA ruptures into the right ventricle or the right ventricular outflow tract, the right ventricular pressure is significantly lower than the aortic pressure in the diastole, and the left-to-right shunt occurs in the entire cardiac cycle, which is mainly in the diastole. When an SVA ruptures into the right atrium, the left-to-right shunt occurs in the entire cardiac cycle due to the low pressure of the right atrium. Numerous shunts cause an increase in the volume load of the right and left ventricles, a compensatory enlargement of the cardiac cavity, and compensatory hypertrophy of the ventricular wall. These outcomes can eventually lead to heart failure and pulmonary hypertension. The hemodynamic changes caused by a small amount of shunt were not evident in our patient.

SVA is usually asymptomatic. Previous reports based on necropsy and cardiac surgery findings estimated that 20% of SVAs were unruptured. Chest pain, palpitations, shortness of breath, and coughing may occur when an SVA ruptures. Acute, large rupture may present with a dramatic onset of marked substernal chest pain, upper abdominal pain, severe dyspnea, and sudden hemodynamic collapse. Symptoms often follow physical stress, with acute dyspnea and chest pain developing after heavy exertion. Regardless of the location of a ruptured aneurysm, resulting in left-to-right or left-to-left shunt, volume overload of the left heart inevitably occurs. Acute symptoms of heart failure may last for hours or days, followed by progressive improvement or worsening of symptoms. Small perforations of aneurysms present more insidiously; patients may remain asymptomatic for months or years until symptoms of congestive heart failure develop. Sudden cardiac death due to unruptured SVA may result from tamponade, myocardial ischemia, conduction disturbances and arrhythmias. During the physical examination, continuous murmurs of the entire cardiac cycle were detected in the auscultation area of the parasternal aorta [6]. Complete right bundle branch block seen in the electrocardiogram in our patient was consistent with a report by Choudhary et al. [7], who reviewed 26 cases of SVA dissecting into the interventricular septum. The differential diagnosis of ventricular cystic masses includes infections secondary to Echinococcus that cause hydatid cysts or Taenia solium that causes cysticercosis, capillary hemangiomas, cystic thrombi, congenital blood cysts, and intracardiac tumors. The clinical context and characteristics of the masses can help establish an accurate diagnosis.

Echocardiography is currently the preferred method for diagnosing ruptured SVA with high accuracy. Specifically, TEE can provide more information due to its high resolution, rendering it valuable for the identification, localization, and differential diagnosis of ruptured aneurysms of the sinus of Valsalva [8]. MRI, contrast aortography, and CT have been used as supplemental or confirmatory tests. Magnetic resonance imaging with multiplanar sequencing is useful for evaluating intracardiac shunts in ruptured SVA.Cardiac catheterization should be used to confirm the diagnosis of the SVA, to evaluate the hemodynamic significance of the rupture, associated cardiac anomalies and, perhaps most importantly, to define coronary anatomy.

The life expectancy of patients with successful SVA repair is close to that of healthy people, with a 10-year survival rate of 90–95% [9]. The need for intervention in SVA is controversial. For a unruptured SVA, concurrent factors should be considered before repair. Intervention is required for outflow tract obstruction, arrhythmia, or infection. Surgical intervention is recommended for a ruptured SVA or an SVA with associated intracardiac abnormalities such as ventricular septal defect or significant aortic valve regurgitation. Surgical repair should also be considered for an unruptured but symptomatic or growing SVA [5–10].

## Conclusion

SVA is a rare congenital or acquired cardiac defect that has been increasingly diagnosed due to improved imaging techniques. Surgical repair is indicated for ruptured or symptomatic SVA.

## Data Availability

Not applicable.

## References

[1] Valsalva AM. Viri Celeberrimi Antonii Mariae Valsalvae. Opera 1740.

[2] Ott DA. Aneurysm of the sinus of Valsalva. Semin Thorac Cardiovasc Surg Pediatr. 10.1053/j.pcsu.2006.02.014 16638563

[3] Card Surg A. 2006: 165–76. 10.1053/j.pcsu.2006.02.014 16638563

[4] Feldman DN, Gade CL, Roman MJ (2005). Ruptured aneurysm of the right sinus of Valsalva. Tex Heart Inst J.

[5] Au WK, Chiu SW, Mok CK, Lee WT, Cheung D, He GW (1998). Repair of ruptured sinus of Valsalva aneurysm: determinants of long-term survival. Ann Thorac Surg.

[6] Takach TJ, Reul GJ, Duncan MJ (1999). Sinus of Valsalva aneurysm or fistula: management and outcome. Ann Thorac Surg.

[7] Feldman DN, Roman MJ (2006). Aneurysms of the sinuses of valsalva. Cardiology.

[8] Choudhary SK, Bhan A, Reddy SC (1998). Aneurysm of sinus of Valsalva dissecting into interventricular septum. Ann Thorac Surg.

[9] McKenney PA, Shemin RJ, Wiegers SE (1992). Role of transesophageal echocardiography in sinus of Valsalva aneurysm. Am Heart J.

[10] Lee JH, Yang ji-Hyuk, Park PW (2021). Surgical Repair of Sinus of Valsalva Aneurysm A 22-Year single-center experience. Thorac Cardiovasc Surg.

